# ATF6 regulates the development of chronic pancreatitis by inducing p53-mediated apoptosis

**DOI:** 10.1038/s41419-019-1919-0

**Published:** 2019-09-10

**Authors:** Lei Zhou, Jie-hui Tan, Rong-chang Cao, Jia Xu, Xue-mei Chen, Zhao-chang Qi, Su-ying Zhou, Su-bing Li, Qi-xin Mo, Zhi-wei Li, Guo-wei Zhang

**Affiliations:** 1 Department of Hepatobiliary Surgery, Nanfang Hospital, Southern Medical University, Guangzhou, China; 2 Department of Pathophysiology, Southern Medical University, Guangzhou, China; 3 Department of Occupational Health and Medicine, Guangdong Provincial Key Laboratory of Tropical Disease Research, School of Public Health, Southern Medical University, Guangzhou, China; 4 The First School of Clinical Medicine, Southern Medical University, Guangzhou, China

**Keywords:** Cell biology, Pathogenesis

## Abstract

Chronic pancreatitis (CP) is a progressive, recurrent inflammatory disorder of the pancreas. Initiation and progression of CP can result from serine protease 1 (PRSS1) overaccumulation and the ensuing endoplasmic reticulum (ER) stress. However, how ER stress pathways regulate the development and progression of CP remains poorly understood. In the present study we aimed to elucidate the ER stress pathway involved in CP. We found high expression of the ER stress marker genes ATF6, XBP1, and CHOP in human clinical specimens. A humanized PRSS1 transgenic mouse was established and treated with caerulein to mimic the development of CP, as evidenced by pathogenic alterations, collagen deposition, and increased expression of the inflammatory factors IL-6, IL-1β, and TNF-α. ATF6, XBP1, and CHOP expression levels were also increased during CP development in this model. Acinar cell apoptosis was also significantly increased, accompanied by upregulated p53 expression. Inhibition of ATF6 or p53 suppressed the expression of inflammatory factors and progression of CP in the mouse model. Finally, we showed that p53 expression could be regulated by the ATF6/XBP1/CHOP axis to promote the development of CP. We therefore conclude that ATF6 signalling regulates CP progression by modulating pancreatic acinar cell apoptosis, which provides a target for ER stress-based diagnosis and treatment of CP.

## Introduction

Chronic pancreatitis (CP) is characterized by persistent inflammation in the pancreas that usually causes irreversible structural damage and severely impairs digestive functions and pancreatic hormone secretion^1,2^. Epidemiological studies have demonstrated that CP is closely associated with many aetiological factors, such as alcohol use, smoking, ischaemia, intraductal obstruction, and calcific stones, as well as genetic factors^2–5^. Long-term CP is a critical risk factor for pancreatic cancer, which is one of the most lethal human malignancies^6^. Despite the clinical application of pancreatic enzyme replacement, therapeutic endoscopy, and surgery, the prevalence of CP remains high, partially due to our poor understanding of its pathogenic mechanisms^7^.

Overaccumulation and misfolding of the serine protease 1 (PRSS1), also known as trypsinogen, have been established as key driving factors for the development of some types of pancreatitis, such as hereditary or idiopathic CP^8,9^. PRSS1 is the principal isoform of trypsinogen secreted by the pancreas. After cleavage into its active form in the small intestine, PRSS1 is responsible for the hydrolysation of proteins^8^. In a transgenic murine model, wild-type or mutant human PRSS1 expression in acinar cells promoted cell apoptosis and spontaneous pancreatitis^10^. However, the pathogenic mechanisms underlying PRSS1 overaccumulation-induced CP, especially the downstream molecular events, are far from fully understood.

Endoplasmic reticulum (ER) stress induced by protein misfolding and overaccumulation in the ER lumen has been documented to be chronically activated in CP and may thus be an important pathogenic mechanism in this disease^11,12^. During ER stress, also known as the unfolded protein response, ER sensors such as ATF6, PERK, and IRE1 become activated and promote the degradation of misfolded proteins, production of molecular chaperones, and cell apoptosis^13,14^. Genetic mutations that rendered PRSS1 more resistant to degradation by the trypsinogen-degrading enzyme chymotrypsin C result in the overaccumulation of misfolded PRSS1 and ER stress during CP pathogenesis^15,16^. In addition, ER stress induced by mutation and misfolding of the carboxyl ester lipase (CEL) protein is also involved in CP development by regulating acinar cell apoptosis^17^. Consistently, pancreatic tissues from patients with CP exhibit significant DNA fragmentation, apoptotic nuclei, and increased p53 expression, as well as substantial alterations in other apoptosis-regulating factors^18^. These observations suggest that ER stress and apoptosis might be involved in the development of CP. How ER stress pathways regulate CP development and progression remains poorly understood.

The present study aimed to elucidate the ER stress mechanism involved in CP pathogenesis. We found that, among ER stress-pathway regulators, ATF6 expression was significantly increased in clinical pancreatic specimens from patients with CP. In a PRSS1 transgenic mouse CP model, knockdown or overexpression of ATF6 affected the severity of CP. We discovered that p53, a master regulator of apoptosis, mediates ATF6-induced acinar cell apoptosis in the CP model. These results suggest, for the first time, that modulating the key molecule ATF6 could prevent the development of CP.

## Results

### CP patients have enhanced ER stress responses

**Fig. 1:**
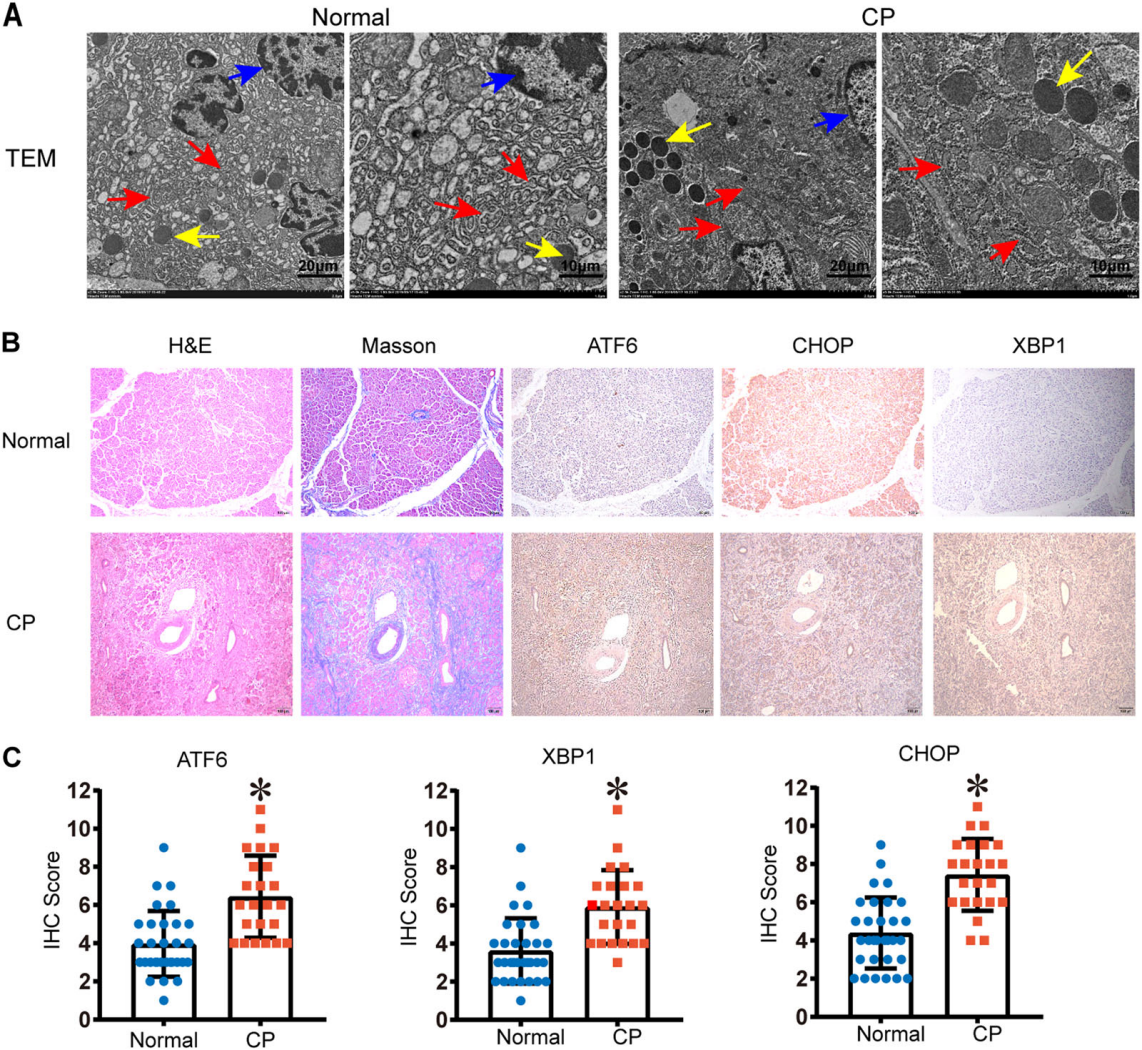
ER destruction in human CP tissues accompanied by high expression of the ER stress-responsive factors ATF6/XBP1/CHOP. **a** The morphology of human normal pancreatic tissue and chronic pancreatitis (CP) tissue was observed by transmission electron microscopy (TEM). Red arrows (↑): endoplasmic reticulum; yellow arrows (↑): zymogen granule; blue arrows (↑): cell nucleus. **b** Haematoxylin and eosin (H&E) and Masson’s trichrome staining of pancreatic tissues and ATF6, CHOP, and XBP1 expression determined by immunohistochemistry (IHC) in CP patients and healthy volunteers. **c** The IHC score was used to quantify the relative expression of ATF6, CHOP, and XBP1. ATF6 activating transcription factor 6, CHOP C/EBP-homologous protein, ER endoplasmic reticulum, XBP1 X-box-binding protein 1. *P < 0.05

To elucidate the signalling pathways by which ER stress activation causes CP, we first aimed to identify the main ER stress effector proteins activated in CP patients. We collected pancreatic tissues from CP patients and healthy volunteers (Supplemental Table S1). Pancreatic tissues from CP patients showed histological and cellular alterations typical of CP: increased collagen in the peri-acinar areas, frequent disappearance and vacuolization of acinar cells, and substantial pancreatic impairment (Fig. 1b). Using transmission electron microscopy, we observed that the ER was broken into fragments and bubbles of different sizes in human CP tissues, which indicated ER stress activation (Fig. 1a). Subsequently, we carried out an immunohistochemistry (IHC) analysis of effector proteins in the ER stress pathways. Compared with those in healthy volunteers, the protein levels of activating transcription factor 6 (ATF6), C/EBP-homologous protein (CHOP), and X-box-binding protein 1 (XBP1) were significantly increased in pancreatic tissues from patients with CP (Fig. 1b, c). These observations suggested that the ER stress-responsive ATF6/XBP1/CHOP axis might be involved in the development of CP.

### ER stress responses are enhanced during CP progression in PRSS1 transgenic mice

**Fig. 2:**
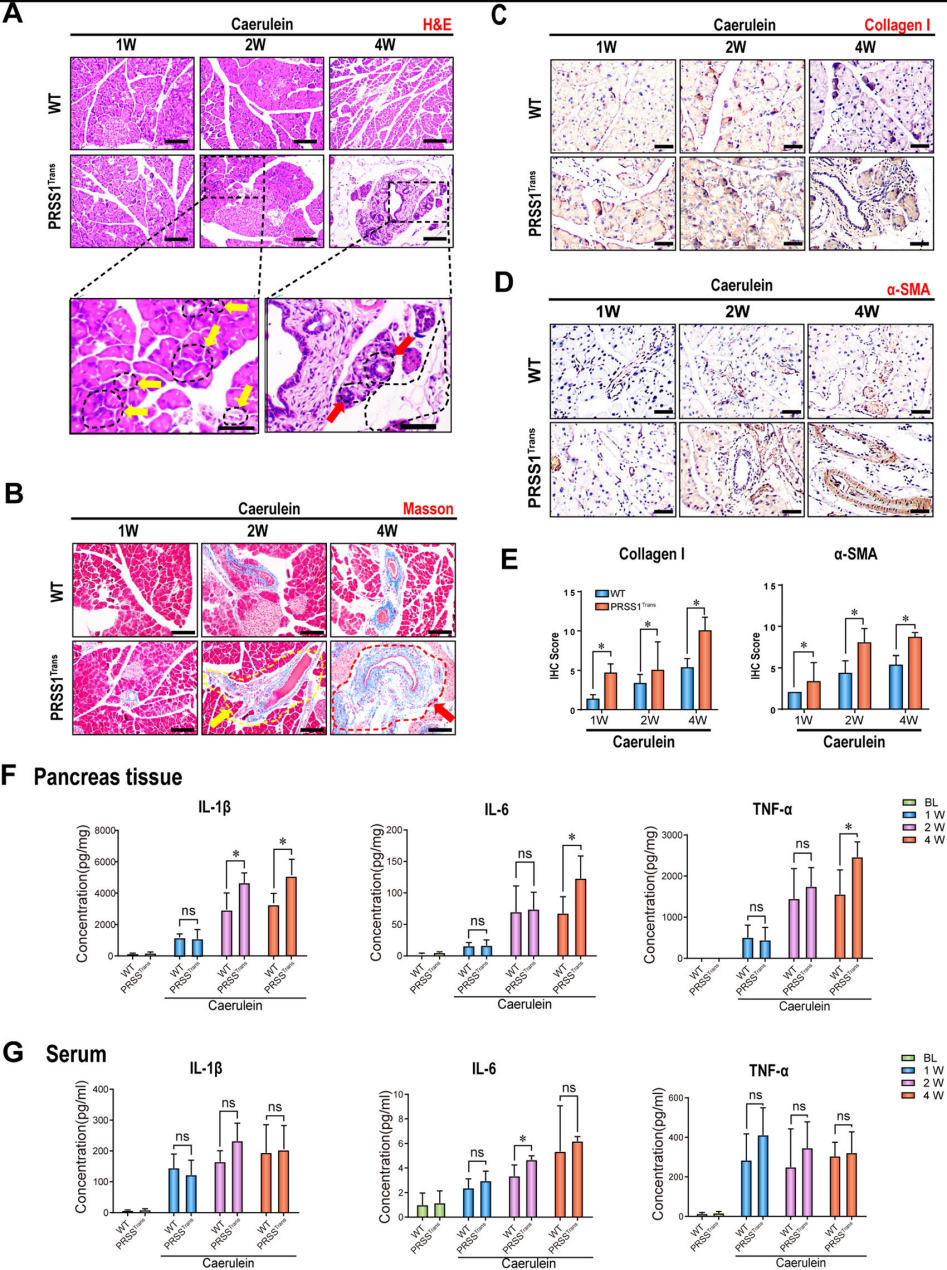
CP model established by caerulein injection in PRSS1 transgenic mice (PRSS^Trans^). **a** Histological evaluation of pancreatic tissues collected from chronic pancreatitis (CP) model mice and wild-type (WT) mice by haematoxylin and eosin (H&E) staining. **b** Analysis of collagen deposition by Masson’s trichrome staining (Red (↑) and yellow arrows (↑) indicate increased fibrosis in pancreatic tissues from PRSS1 transgenic mice at 2 and 4 weeks post-caerulein injection in pancreatic tissues from PRSS1 transgenic mice, respectively.) and **c** collagen I levels and **d** α-smooth muscle actin (α-SMA) levels by immunohistochemistry (IHC) in pancreatic tissues from CP model mice and WT mice sacrificed 1, 2, or 4 weeks post-caerulein injection. **e** IHC histological scores of pancreatic tissues in CP model mice after finishing caerulein treatment. The levels of IL-1β, IL-6, and TNF-α in pancreatic tissues (**f**) and serum (**g**) from WT and PRSS1 transgenic mice treated with caerulein for CP induction, as determined by ELISA and quantitative RT-PCR. PRSS1 serine protease 1, α-SMA α-smooth muscle actin, IL-1β interleukin 1β, IL-6 interleukin 6, TNF-α tumour necrosis factor-α, BL baseline, W weeks, ns no significant difference; *P < 0.05

To further study the role of the ATF6/XBP1/CHOP axis in CP, we established a mouse CP model by treating humanized *PRSS1* transgenic mice with caerulein (Supplemental Fig. S1B). We found that pancreas size in the caerulein-treated PRSS1 transgenic mice decreased substantially over time but this effect was not seen in wild-type mice (Supplemental Fig. S2). Four weeks after caerulein injection, we observed substantial disappearance of pancreatic parenchymal cells and increased fibrosis in pancreatic tissues from PRSS1 transgenic mice (Fig. 2a, b). Collagen I and α-smooth muscle actin (α-SMA) deposition were also significantly higher in pancreatic tissues from caerulein-treated PRSS1 transgenic mice than wild-type mice (Fig. 2c–e). Additionally, we detected the expression of several key players in inflammatory responses in the mouse CP model. We found that levels of the inflammatory factors interleukin 1β (IL-1β), interleukin 6 (IL-6), and tumour necrosis factor-α (TNF-α) were substantially increased in mouse pancreatic tissues after caerulein injection. Importantly, significantly greater increases in these inflammatory factors were observed in pancreatic tissues of PRSS1 transgenic mice than wild-type mice (Fig. 2f), but in the serum no significant differences were observed between wild-type and PRSS1 transgenic mice except IL-6 at the 2 weeks time point (Fig. 2g). These pathological and molecular data show that PRSS1 transgenic mice can be used as an ideal animal CP model for subsequent analysis. More severe CP manifestation observed in PRSS1 transgenic mice than in wild-type mice confirmed PRSS1 overexpression as a driving force for the pathogenesis of CP.

**Fig. 3:**
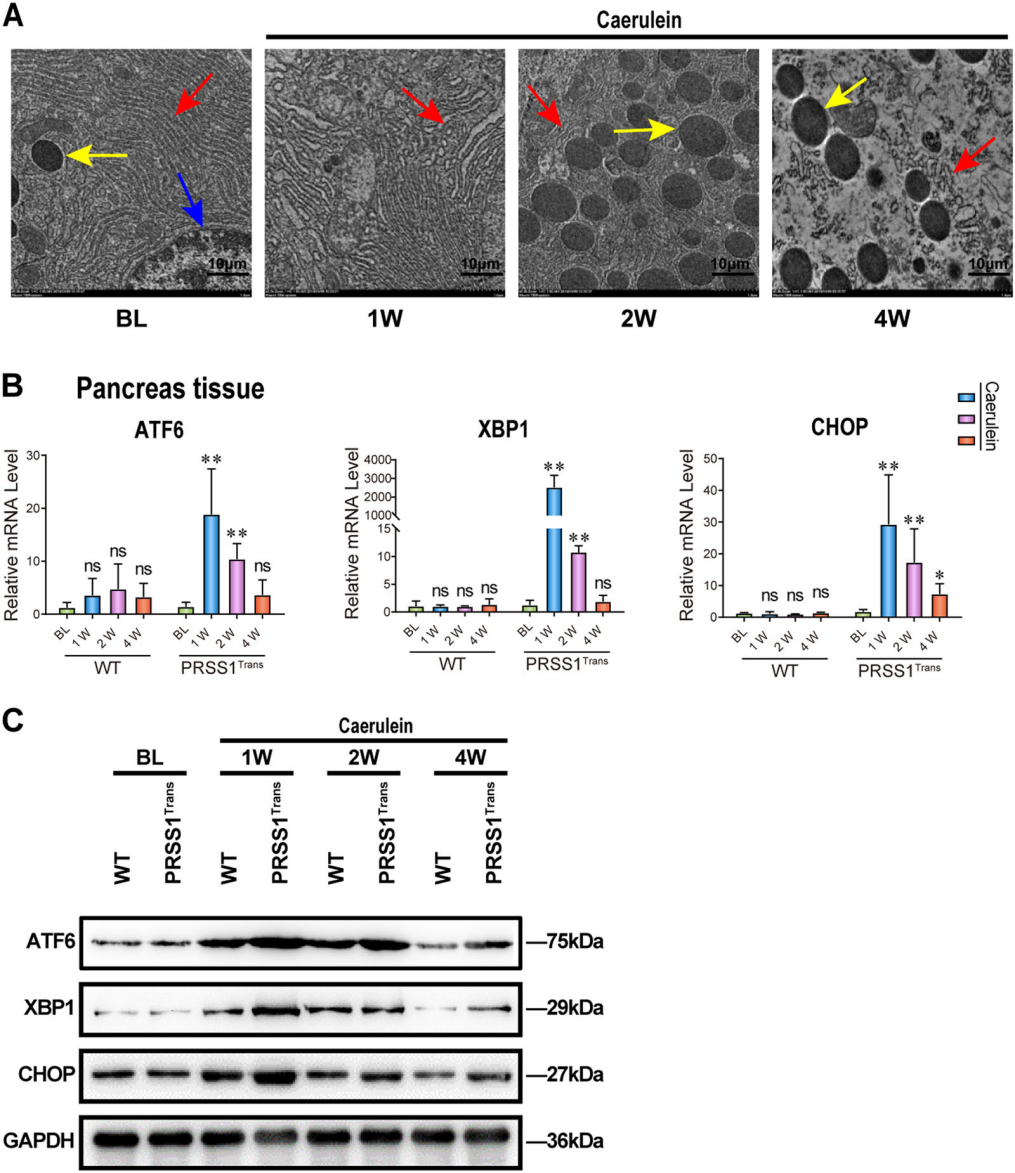
ER stress responses in PRSS1 transgenic mice treated with caerulein. **a** Morphological changes in the endoplasmic reticulum (ER) in response to caerulein treatment in PRSS1 transgenic mice were determined by transmission electron microscopy. Red arrows (↑): endoplasmic reticulum; yellow arrows (↑): zymogen granule; blue arrows (↑): cell nucleus. ATF6, XBP1, and CHOP gene (**b**) and protein (**c**) levels in pancreatic tissues, as determined by quantitative RT-PCR and western blotting, respectively. ATF6 activating transcription factor 6, XBP1 X-box-binding protein 1, CHOP C/EBP-homologous protein, GAPDH glyceraldehyde-3-phosphate dehydrogenase, ns no significant difference, WT wild-type, BL baseline, W weeks, ns no significant difference; *P < 0.05 and **P < 0.01

Next, we observed a gradual increase in ER destruction in the pancreatic tissues in the mouse CP model, indicating an increased activation of the ER stress response (Fig. 3a). To confirm what we had observed in human samples, we measured the expression of the same three key players in ER stress responses in the mouse CP model. *Atf6, Xbp1* and *Chop* were significanlty induced in PRSS1-overexpressing mice treated with caerulein compared to wild-type mice treated with caerulein, both at the mRNA and protein levels (Fig. 3b, c). These results demonstrate significant activation of the ER stress-responsive ATF6/XBP1/CHOP axis during CP progression in PRSS1 transgenic mice according with the results observed in patients with CP.

### Acinar cell apoptosis and p53 levels are increased in the mouse CP model

**Fig. 4:**
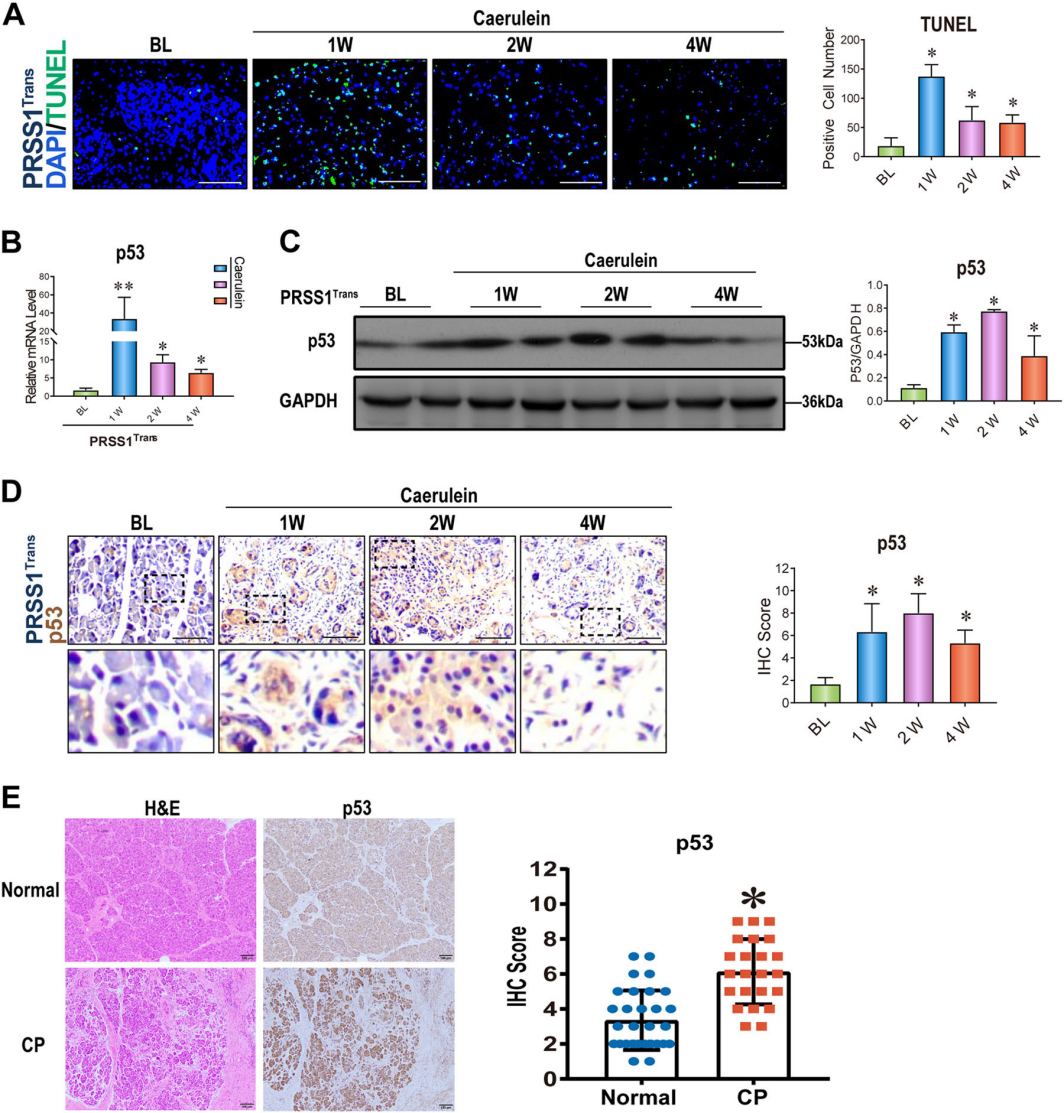
Acinar apoptosis and p53 expression in CP. **a** Transferase-mediated d-UTP nick-end-labelling (TUNEL) assay in PRSS1 transgenic mice treated with caerulein for 1, 2, and 4 weeks. **b** p53 mRNA levels in pancreatic tissues from PRSS1 transgenic mice treated with caerulein to induce chronic pancreatitis (CP). Mice were sacrificed 1, 2, and 4 weeks after treatment, and the mRNA levels were determined by quantitative RT-PCR. p53 protein levels in pancreatic tissues from PRSS1 transgenic mice treated with caerulein by Western blotting (**c**) and immunohistochemistry (IHC) (**d**). **e** Haematoxylin and eosin (**h**, **e**) and p53 IHC in CP patients and healthy volunteers. BL baseline, W weeks; *P < 0.05

To investigate how ATF6 induction regulates CP development, we measured apoptosis, which had a close relationship with ER stress, in the mouse CP model. A transferase-mediated d-UTP nick-end-labelling (TUNEL) assay showed that numbers of apoptotic cells in PRSS1 mice pancreatic tissues were remarkably increased 1 and 2 weeks after caerulein treatment. This effect lessened over time, along with a decrease in the number of pancreatic acinar cells by week 4 (Fig. 4a). To investigate the mechanism underlying apoptosis induction in CP, we measured levels of p53, a master regulator of apoptosis, in the CP model. Caerulein treatment significantly induced p53 mRNA and protein levels in pancreatic tissues from PRSS1 transgenic, with the highest levels observed 1 and 2 weeks after treatment, respectively (Fig. 4b, c). These results were confirmed by IHC that also showed a progressive disappearance of pancreatic parenchymal cells and appearance of fibrosis in pancreatic tissues (Fig. 4d). Consistently, p53 expression was higher in patients with CP than individuals without CP (Fig. 4e). These results suggest that acinar cell apoptosis and p53 might be involved in CP.

### ER stress regulates p53 during CP progression

**Fig. 5:**
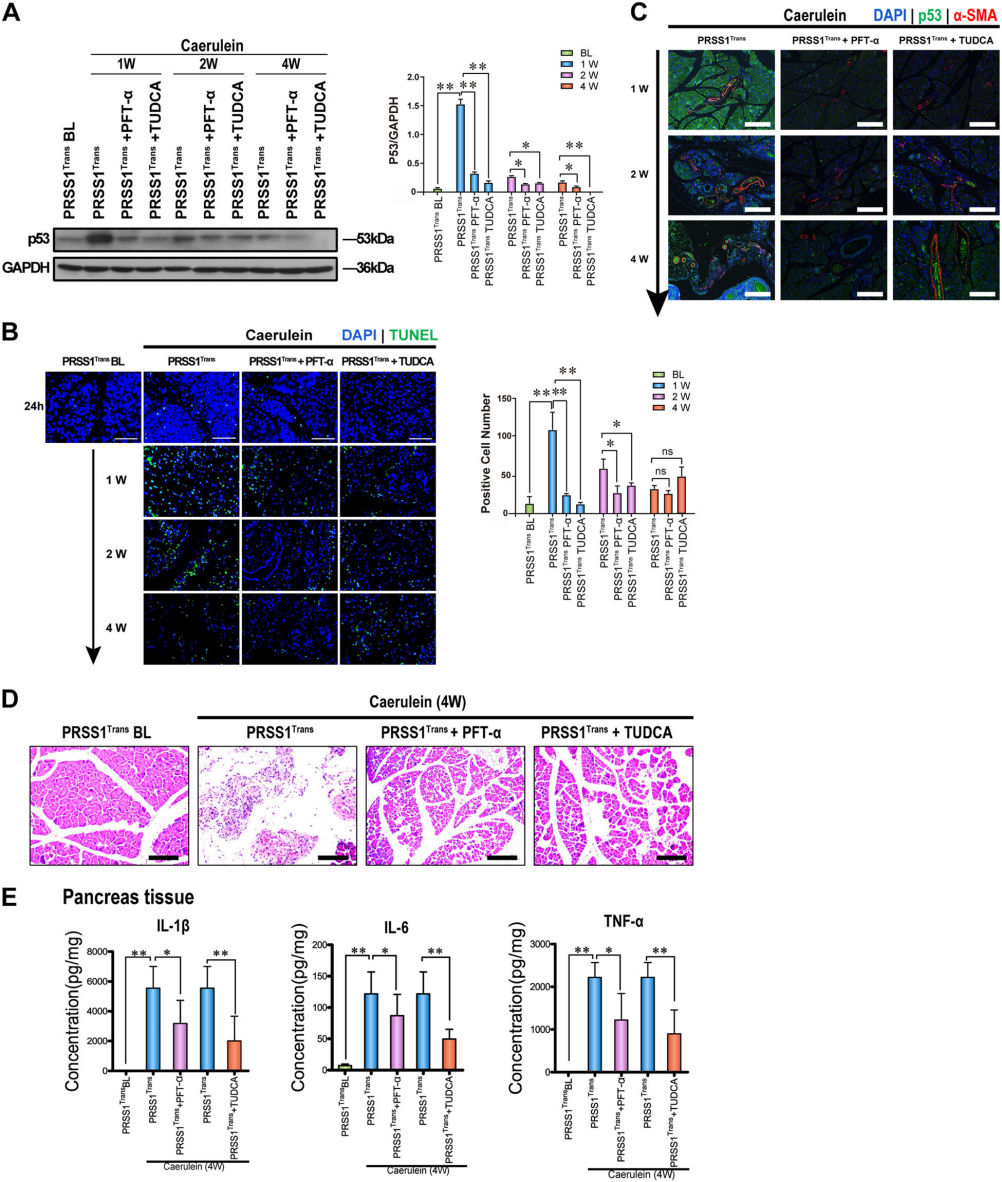
Regulation of ER stress by p53 during CP progression in PRSS1 transgenic mice. **a** The expression of p53 in PRSS1 transgenic mice treated with pifithrin-α (PFT-α; a p53 inhibitor) and tauroursodeoxycholic acid (TUDCA; an ER stress inhibitor) at 1, 2, and 4 weeks after treatment with caerulein. **b** Transferase-mediated d-UTP nick-end-labelling (TUNEL) assay showing acinar cell apoptosis in caerulein-treated PRSS1 transgenic mice after PFT-α and TUDCA treatments. **c** p53 and α-smooth muscle actin (α-SMA) protein expression levels in pancreatic tissues from caerulein-treated PRSS1 transgenic mice with p53 inhibition by PFTα. **d** Pathological alteration of pancreatic tissues from caerulein-treated PRSS1 transgenic mice with p53 inhibition by PFTα. Tissues were stained with haematoxylin and eosin (H&E). **e** Expression of IL-1β, IL-6, and TNF-α in pancreatic tissues from caerulein-treated PRSS1 transgenic mice treated with PFT-α and TUDCA. IL-1β interleukin 1β, IL-6 interleukin 6, TNF-α tumour necrosis factor-α, BL baseline, W weeks, ns no significant difference; *P < 0.05 and **P < 0.01

To determine whether p53 induction during CP progression was related to ER stress activation, we inhibited p53 expression and the ER stress response in the mouse CP model using pifithrin-α (PFT-α) and tauroursodeoxycholic acid (TUDCA), respectively. We observed that p53 expression was significantly suppressed by both PFT-α and TUDCA treatments, indicating that ER stress could effectively regulate p53 expression during CP pathogenesis (Fig. 5a). Furthermore, acinar cell apoptosis in PRSS1 transgenic mice treated with caerulein was significantly suppressed by both PFT-α and TUDCA treatments, accompanied by reduced parenchymal cell loss and improved pancreatic structural integrity (Fig. 5b).

Moreover, α-SMA levels in PRSS1 transgenic mice treated with caerulein increased, while no such alteration in α-SMA levels was observed when PRSS1 transgenic mice were treated with the p53 or ER stress response inhibitors (Fig. 5c). Four weeks after caerulein treatment, inhibition of p53 expression or ER stress appeared to prevent pancreatic cell disappearance, tissue destruction, and fibrosis in the CP mouse model (Fig. 5d). In addition, we found that inhibiting p53 expression or ER stress response decreased IL-6, IL-1β, and TNF-α protein levels in pancreatic tissues from the CP model mice 4 weeks after caerulein treatment compared with those in mice without p53 or ER stress response inhibition (Fig. 5e). These results show that ER stress-induced p53 expression could modulate the progression of CP in PRSS1 transgenic mice.

### ATF6 promotes p53 expression, inflammation, and acinar cell apoptosis in vitro

**Fig. 6:**
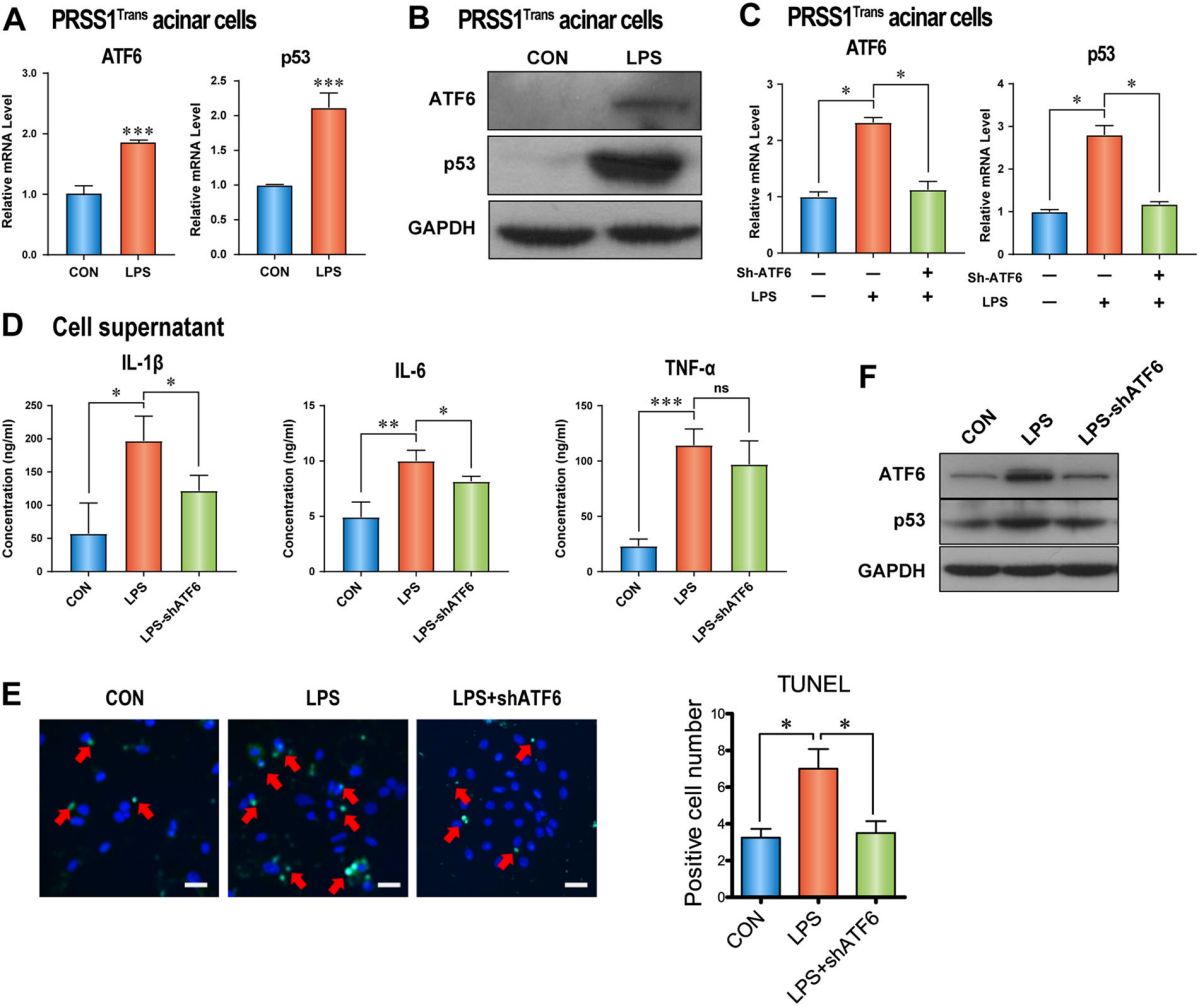
Regulation of p53 expression and CP progression by ATF6 in PRSS1 acinar cells. The expression levels of ATF6 and p53 in response to lipopolysaccharide (LPS) induction in primary acinar cells isolated from PRSS1 transgenic mice were determined by quantitative RT-PCR (**a**) and western blotting (**b**). ATF6 expression (**c**), inflammatory factors (**d**), and cell apoptosis (**e**) measured by quantitative RT-PCR, ELISA, and transferase-mediated d-UTP nick-end-labelling (TUNEL) assays, respectively. **f** Expression of ATF6 and p53 in LPS-induced ATF6-suppressed cells. shATF6 shATF6 virus, CON control group, ATF6 activating transcription factor 6, ns no significant difference; *P < 0.05 and **P < 0.01

To analyze whether ER stress response regulation of p53 levels occurred via ATF6, primary acinar cells were isolated from PRSS1 transgenic mice and treated with lipopolysaccharide (LPS). We observed that ATF6 and p53 expression was induced by LPS in primary acinar cells (Fig. 6a, b). The increase in p53 expression in primary acinar cells induced by LPS treatment was greatly suppressed by ATF6-interfering adenovirus (shATF6) (Fig. 6c). Additionally, inhibition of ATF6 expression resulted in significant decreases in IL-6 and IL-1β levels, but not in TNF-α, in cell culture media from LPS-treated primary acinar cells (Fig. 6d). The apoptosis of primary acinar cells, which was promoted by LPS treatment, was also suppressed by ATF6 inhibition (Fig. 6e). Moreover, LPS-induced p53 expression in acinar cells from PRSS1 transgenic mice was substantially decreased by ATF6-interfering adenovirus (Fig. 6f). These in vitro results show that ATF6 promotes p53 expression, inflammation, and acinar cell apoptosis.

### ATF6 promotes p53 expression and inflammation in vivo

**Fig. 7:**
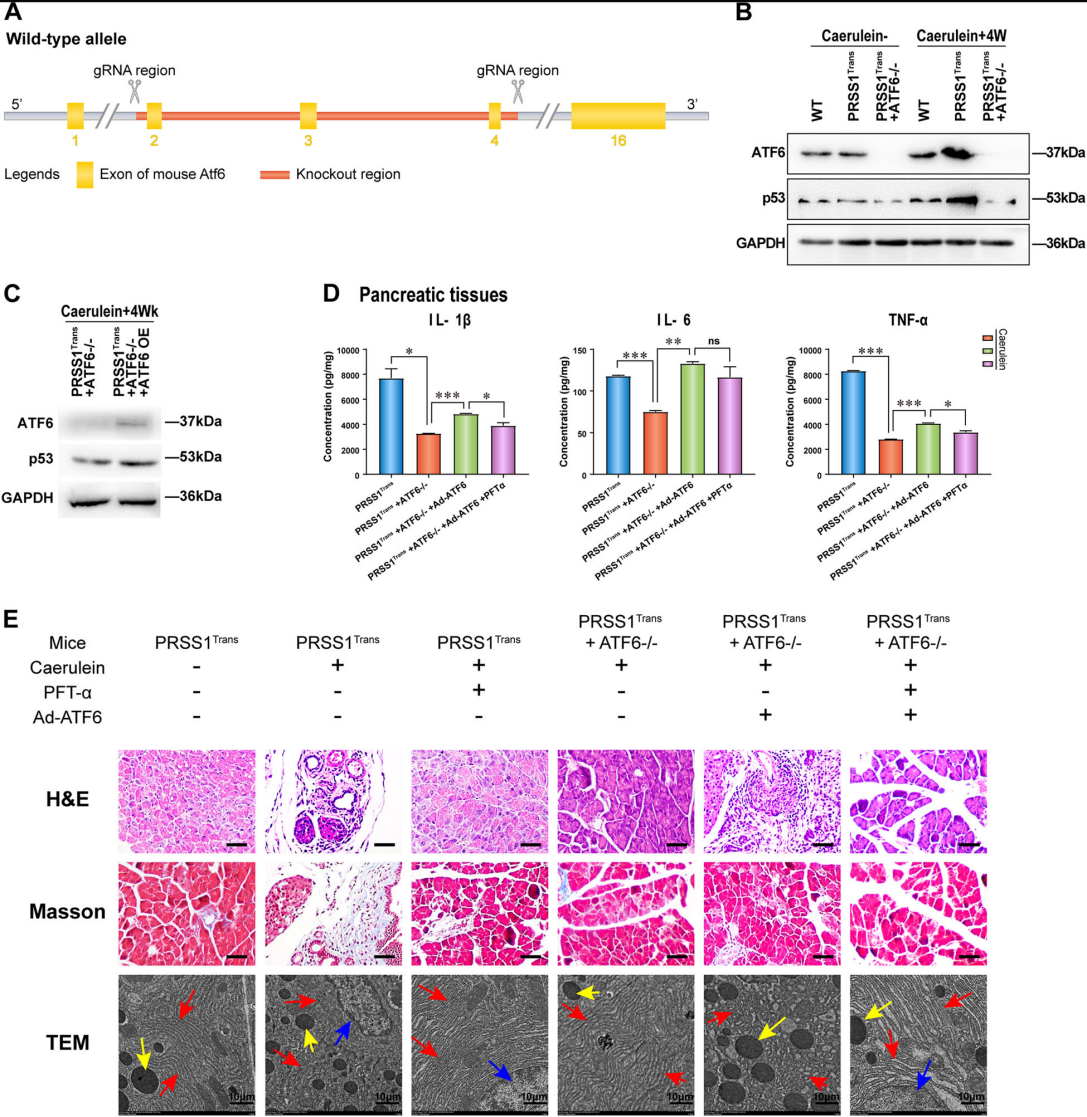
Regulation of p53 expression and CP progression by ATF6 in the PRSS1 CP model. **a** Schematic diagram of ATF6 knockout mice. **b** The expression levels of ATF6 and p53 were determined by western blotting. ATF6 and p53 protein levels (**c**) and IL-6, IL-1β, and TNF-α pancreatic tissues concentrations (**d**) in PRSS1 transgenic mice with ATF6 knockout and overexpression after induction of chronic pancreatitis (CP) for 4 weeks. **e** Haematoxylin and eosin (**h**, **e**) and Masson’s trichrome staining and transmission electron microscopy (TEM) images showing the pathological changes in ATF6-deficient PRSS1 transgenic mice and ATF6 deficiency in ATF6-rescued PRSS1 transgenic mice. Red arrows (↑): endoplasmic reticulum; yellow arrows (↑): zymogen granule; blue arrows (↑): cell nucleus. ATF6 activating transcription factor 6, OE overexpression, Ad-ATF6 Ad-ATF6 virus, PFT-α pifithrin-α, IL-1β interleukin 1β, IL-6 interleukin 6, TNF-α tumour necrosis factor-α. ns no significant difference, W week, *P < 0.05 and **P < 0.01

For further confirmation of the effect of ATF6 on p53 expression and CP pathogenesis, PRSS1 transgenic mice were crossed with ATF6 knockout mice (Fig. 7a). Four weeks after caerulein treatment, p53 expression in PRSS1 transgenic ATF6 knockout mice was lower than that in wild-type mice and PRSS1 transgenic mice treated with caerulein (Fig. 7b). In contrast, ATF6 gene overexpression increased p53 expression in PRSS1 transgenic ATF6^−/−^ mice 4 weeks after caerulein treatment (Fig. 7c). In addition, we observed that levels of the inflammatory factors IL-6, IL-1β, and TNF-α in the pancreatic tissues of PRSS1 transgenic mice after CP induction by caerulein were significantly reduced by ATF6 gene knockout but rescued by ATF6 overexpression to levels comparable with those in PRSS1 transgenic mice after CP induction (Fig. 7d).

Finally, PRSS1 transgenic ATF6 knockout mice and ATF6 complemented mice were treated with PFT-α to further explore the involvement of the ER stress response and p53 in CP pathogenesis. Haematoxylin and eosin (H&E) and Masson’s trichrome staining revealed that ATF6 knockout and p53 inhibition ameliorated oedema and acinar deformation and decreased inflammatory cell infiltration and collagen accumulation in PRSS1 transgenic mice (Fig. 7e). Recovery of the ER network was observed in mice with p53 inhibition and ATF6 deficiency, while these effects were attenuated after ATF6 expression was restored (Fig. 7e). Combined, these in vivo results show that ATF6 modulates the progression of CP by regulating p53.

## Discussion

CP remains a troublesome disease, usually with unsatisfactory therapeutic effects in the clinic, because its pathogenesis is unclear. In recent decades, several theories have emerged to explain the pathogenesis of CP, including oxidative stress, toxic metabolic effects, immune responses, ER stress, and necrosis-fibrosis^19,20^. The ER is an important organelle involved in cell metabolism and is very rich in pancreatic acinar cells. At the beginning of this study, we found that the ER structure was destroyed and that expression of the ER stress proteins ATF6, XBP1, and CHOP was increased in tissues from CP patients. These phenomena caused us to focus our research on ER stress in CP. As such, we constructed a humanized PRSS1 mouse model that mimicked the development of CP, and found that apoptosis and p53 expression were increased during CP progression in this model. Finally, we determined the relationship between ATF6 and p53 during CP progression with in vivo and in vitro experiments. We demonstrate for the first time that modulating the key molecule ATF6 can prevent the development of CP by regulating p53-mediated apoptosis.

The discovery that PRSS1 overactivation is associated with CP development has been regarded as an essential step forward in understanding the molecular mechanisms of CP pathogenesis in past decades^5^. Additionally, the ER stress response caused by digestive enzymes is considered a critical disease risk factor for CP progression^12,15,21,22^. Previous studies have shown that PRSS1 protein misfolding contributes to the development and progression of CP through inducing ER stress responses^15^, suggesting a protein misfolding-dependent pathway for CP onset and progression^12^. Autophagy-related protein 7 (ATG7)^23^ and kinase α (IKKα)^24^ were reported to modulate CP progression through ER stress, but research on these upstream processes did not demonstrate how downstream ER stress regulates acinar injury. In trypsinogen-7 knockout mice, upregulation of the unfolded protein response components GRP78 and XBP1 was found in CP^11^. Our study also supported the upregulation of ER stress responses (ATF6/CHOP/XBP1) in pancreatic tissues from CP patients. Furthermore, our animal CP model (humanized PRSS1 mice treated with caerulein) also exhibited significantly increased expression of the key ER stress pathway genes ATF6, XBP1, and CHOP. These results further support the hypothesis that the ER stress response pathway exerts critical regulatory roles in CP pathogenesis, which supports further functional research about the role of ER stress in CP.

In addition to its crucial role in pancreatic exocrine function (acinar cells), ER stress is fundamentally involved in β-cell dysfunction. In a rat diabetic model established with a high-fat diet, NAD-dependent deacetylase sirtuin-3 (SIRT3; an important regulator of cell metabolism) inactivated ATF6 and CHOP and protected pancreatic β-cells from ER stress-mediated apoptosis^25^. Recently, a study of atherosclerosis showed that laminar flow protected ER stress-induced cleaved forms of PARP-1 and caspase-3 and inhibited the ER stress-induced p-eIF2α, ATF4, CHOP, spliced XBP-1, ATF6, and JNK pathways to protect endothelial cells from apoptosis^26^. In addition to its role in benign diseases, the ER stress pathway is involved in tumour initiation and progression in pancreatic cancer^27^ and hepatocellular carcinoma^28^; in cholangiocarcinoma, a chemotherapeutic drug was shown to inhibit cholangiocarcinoma proliferation and induce caspase-dependent apoptosis through the ATF6 and XBP1 pathway^29^. From the above findings, we can conclude that, in many diseases, ER stress plays a role by regulating apoptosis.

Due to the close relationship between ER stress and apoptosis, we measured pancreatic acinar cell apoptosis and found that apoptosis was significantly increased in caerulein-treated PRSS1 transgenic mice. p53, a master regulator of cell apoptosis^30^, thus attracted our attention. Moreover, previous investigations have revealed significantly increased expression of the p53 gene during CP pathogenesis^18^. Additionally, during folliculogenesis in mouse granulosa cells, ATF6 plays a modulatory role in proliferation and differentiation by regulating apoptosis and the cell cycle^31^. In the CP mouse model and in tissues from CP patients, we observed that p53 protein expression was significantly increased. Therefore, we speculated that a close interaction existed between ATF6 and p53, and their specific pathogenic functions should be clarified.

Currently, the regulatory mechanism of ATF6 and p53 is still unclear. Previous studies have suggested that ATF6 and CHOP play a key role in NO-mediated apoptosis in macrophages^32^. Induction of the unfolded protein response enhances interactions between ribosomal proteins (rpL5, rpL11, and rpL23) and Hdm2 in a PERK-dependent manner. Interaction with ribosomal proteins results in inhibition of the Hdm2-mediated ubiquitination and degradation of p53^33^. To clarify the regulatory relationship between ATF6 and p53, we inhibited ATF6 and p53, which substantially suppressed pancreatic acinar cell apoptosis and the inflammation in the mouse CP model. Moreover, ATF6 knockdown downregulated p53 expression, accompanied by reduced apoptosis and acinar injury in CP; these results were confirmed in recovery experiments.

However, the exact mechanism how ATF6 promote P53 expression, such as direct binding or not, is unclear and deserves further investigation. The effects of ATF6 on CP pathogenesis might also regulate other signalling pathways. So, the work of seeking the ATF6 downstream molecules by RNA and protein combining screening techniques is on the way.

In summary, by using an ideal CP model, we figured out the regulatory relationship between ATF6 and pancreatic acinar cell apoptosis mediated by p53 during CP progression. We hope these results may help broaden our insight to many other diseases and provide a hint to more studies on ER stress.

## Materials and methods

### Patient cohort

Pancreatic tissues were collected from 23 CP patients (mean age = 44.57 ± 19.79) and 31 patients with benign pancreatic tumours or peritumoural normal pancreatic tissues (mean age = 43.13 ± 13.93) as controls (Supplementary Table S1). All patients were registered at the Department of Hepatobiliary Surgery in Nanfang Hospital, Southern Medical University in Guangzhou, China between 2014 and 2018. The research procedures were approved by the Ethics Committee of Southern Medical University, and written informed consent was obtained from each patient before the study.

### Establishment of PRSS1 transgenic mice, ATF6 knock out mice and ATF6 adenovirus

Humanized *PRSS1* transgenic mice and ATF6 knock mice were constructed. ATF6 adenovirus including ATF6 overexpression, shATF6 for ATF6 inhibition and negative control were also constructed for functional research. Please see the Supplementary Methods for more details.

### Animal studies

Healthy male C57BL/6 mice aged 5–6 weeks were used in this study. All mice were maintained in standard experimental cages at 24 ± 2 °C under a 12 h light/dark cycle and supplied with standard laboratory animal chow and water ad libitum. The experimental operations were performed with approval from the Institutional Animal Care and Use Committee of Southern Medical University.

### CP induction and treatment

To establish a CP animal model, male transgenic mice were intraperitoneally injected with 15 μg/mL caerulein dissolved in phosphate-buffered saline at 50 μg/kg each hour for 8 h. Subsequent replicates of the caerulein injection are shown in Fig. S1B. Please see the Supplementary Methods for more details.

### IHC and immunofluorescence assays

H&E staining and IHC analysis of pancreatic tissue slides for ATF6, CHOP, XBP-1, collagen I, α-SMA, and p53 were performed according to a previously described protocol^34^. For experimental details, please refer to the Supplementary Methods.

### Masson’s trichrome staining

The collagen fibre content of pancreatic tissues was determined using a Masson’s Trichrome Stain Kit (G1340; Solarbio Science & Technology, Beijing, China) according to the manufacturer’s instructions. For experimental details, please refer to the Supplementary Methods.

### Quantitative RT-PCR

Quantitative RT-PCR was performed in this study to detect gene mRNA levels in primary acinar cells or pancreatic tissues. The sequences of the primers used are listed in Table S2. Please see the Supplementary Methods for more details.

### Western blotting

Total protein was extracted from the mouse pancreatic tissues. Primary antibodies used in this study include anti-ATF6, anti-CHOP, anti-XBP-1, anti-p53, and anti-GAPDH. The protein abundance was evaluated by immunoblotting with at least three biological replicates. Please refer to the Supplementary Methods for more details.

### TUNEL assay

Cell apoptosis in mouse pancreatic tissues was analyzed using the TUNEL method with a DNA Fragmentation Imaging Kit (Sigma Aldrich) as instructed by the manufacturer. Please refer to the Supplementary Methods for more details.

### Statistical analysis

Statistical analysis was carried out using GraphPad Prism 5.0 and SPSS 18.0 software, and data are presented as the mean ± the standard error of the mean. Significant differences between two groups were analyzed by Student’s *t*-test, and one-way analysis of variance was performed to investigate the differences among more than two groups. Significant differences were defined by a *P* value of <0.05.

## Supplementary information

Figure S1

Figure S2

Table S1

Table S2

Supplementary Methods

Supplementary figure legends
