# Supplementary figures and images for: ATF6 regulates the development of chronic pancreatitis by inducing p53-mediated apoptosis
Source: Cell Death Dis. 2019 Sep 10;10(9):662. doi: 10.1038/s41419-019-1919-0 (PMC6737032; doi:10.1038/s41419-019-1919-0)

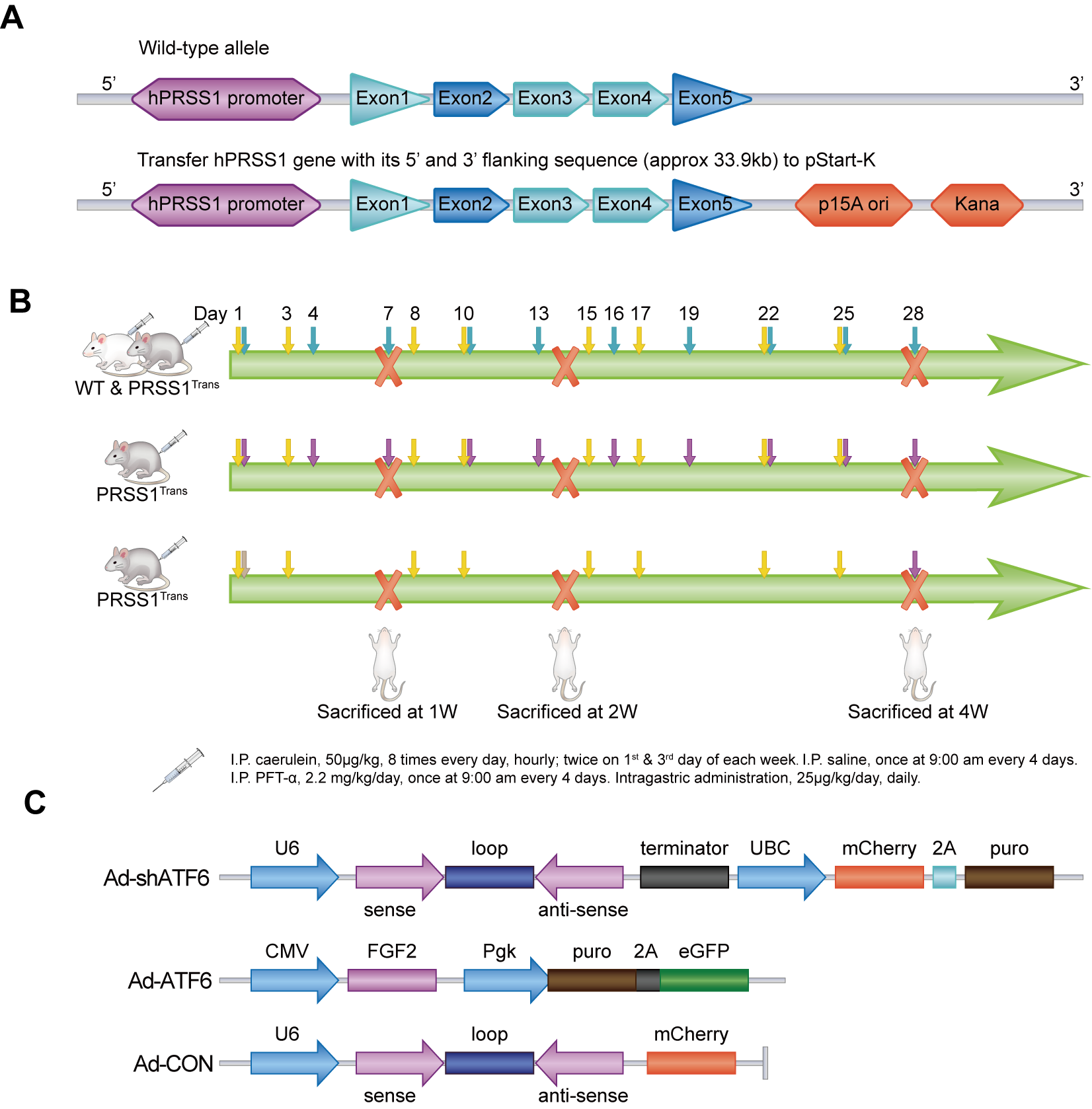

Supplement: Supplementary file 1 — Figure S1 [file 41419_2019_1919_MOESM1_ESM.tif]

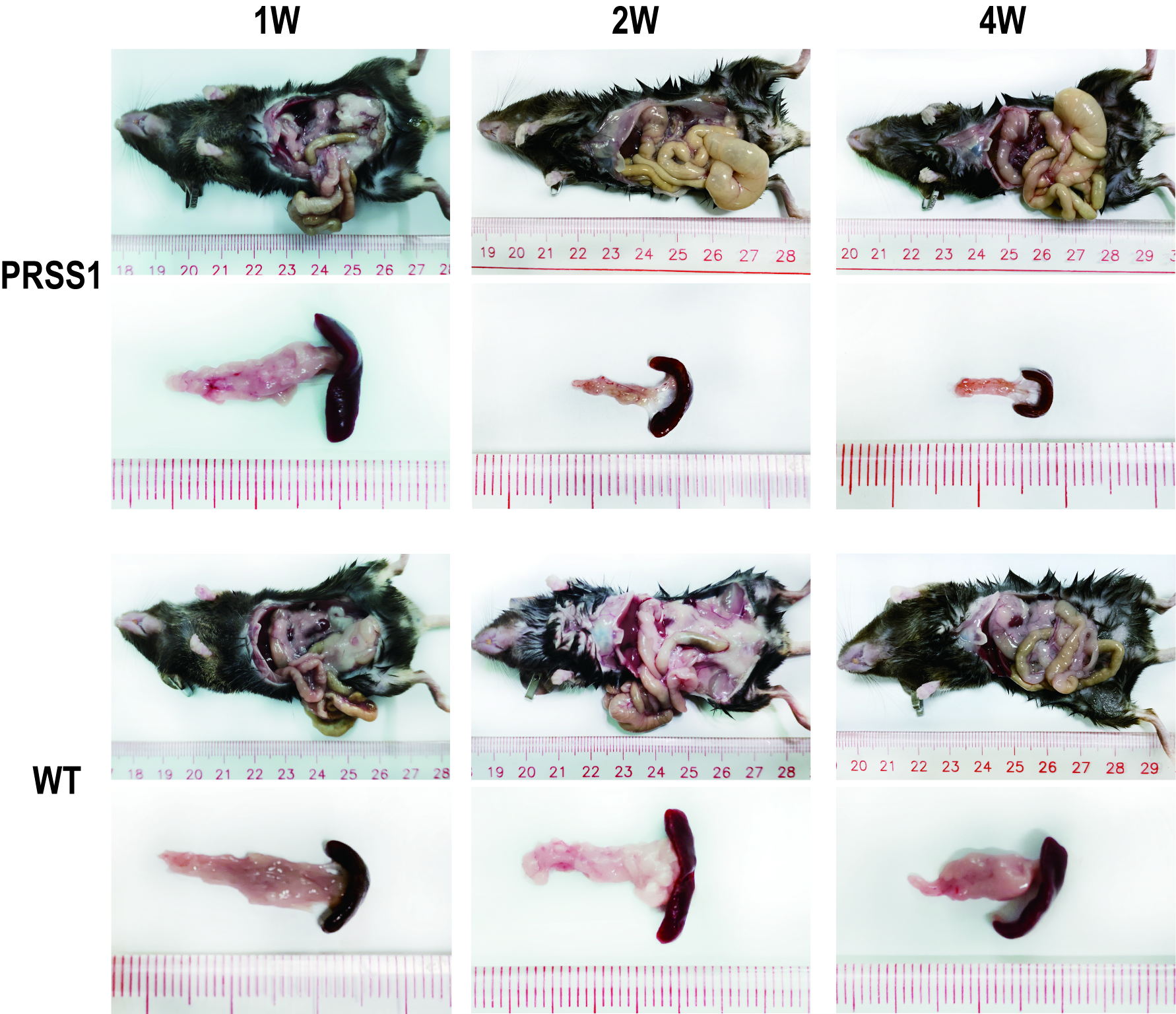

Supplement: Supplementary file 2 — Figure S2 [file 41419_2019_1919_MOESM2_ESM.tif]
