# Supplementary material for: ATF6 regulates the development of chronic pancreatitis by inducing p53-mediated apoptosis
Source: Cell Death Dis. 2019 Sep 10;10(9):662. doi: 10.1038/s41419-019-1919-0 (PMC6737032; doi:10.1038/s41419-019-1919-0)
Supplement: Supplementary file 3 — Table S1 [file 41419_2019_1919_MOESM3_ESM.doc]

**Table S1. Clinical characteristic data of patients and normal controls**

| **No.** | **age** | **Gender** | **Disease** | **Group** |
| --- | --- | --- | --- | --- |
| 1 | 27 | male | Pancreas benign tumer | Normal |
| 2 | 63 | male | Pancreas benign tumer | Normal |
| 3 | 40 | female | Peritumoral | Normal |
| 4 | 52 | male | Pancreas benign tumer | Normal |
| 5 | 28 | male | Pancreas benign tumer | Normal |
| 6 | 30 | female | Pancreas benign tumer | Normal |
| 7 | 32 | male | Pancreas benign tumer | Normal |
| 8 | 56 | male | Peritumoral | Normal |
| 9 | 33 | female | Pancreas benign tumer | Normal |
| 10 | 28 | male | Pancreas benign tumer | Normal |
| 11 | 36 | female | Pancreas benign tumer | Normal |
| 12 | 61 | female | Peritumoral | Normal |
| 13 | 59 | male | Peritumoral | Normal |
| 14 | 29 | female | Pancreas benign tumer | Normal |
| 15 | 35 | male | Peritumoral | Normal |
| 16 | 18 | female | Pancreas benign tumer | Normal |
| 17 | 66 | female | Peritumoral | Normal |
| 18 | 39 | male | Pancreas benign tumer | Normal |
| 19 | 43 | male | Peritumoral | Normal |
| 20 | 44 | female | Peritumoral | Normal |
| 21 | 48 | male | Peritumoral | Normal |
| 22 | 27 | male | Pancreas benign tumer | Normal |
| 23 | 43 | female | Peritumoral | Normal |
| 24 | 70 | male | Peritumoral | Normal |
| 25 | 69 | female | Peritumoral | Normal |
| 26 | 43 | male | Pancreas benign tumer | Normal |
| 27 | 56 | female | Peritumoral | Normal |
| 28 | 34 | male | Pancreas benign tumer | Normal |
| 29 | 42 | female | Peritumoral | Normal |
| 30 | 49 | male | Peritumoral | Normal |
| 31 | 37 | female | Pancreas benign tumer | Normal |
| 32 | 26 | female | Chronic pancreatitis | CP |
| 33 | 34 | male | Chronic pancreatitis | CP |
| 34 | 42 | female | Chronic pancreatitis | CP |
| 35 | 20 | male | Chronic pancreatitis | CP |
| 36 | 39 | male | Chronic pancreatitis | CP |
| 37 | 34 | male | Chronic pancreatitis | CP |
| 38 | 57 | male | Chronic pancreatitis | CP |
| 39 | 75 | female | Chronic pancreatitis | CP |
| 40 | 31 | male | Chronic pancreatitis | CP |
| 41 | 43 | male | Chronic pancreatitis | CP |
| 42 | 42 | male | Chronic pancreatitis | CP |
| 43 | 38 | male | Chronic pancreatitis | CP |
| 44 | 42 | male | Chronic pancreatitis | CP |
| 45 | 78 | female | Chronic pancreatitis | CP |
| 46 | 12 | male | Chronic pancreatitis | CP |
| 47 | 69 | male | Chronic pancreatitis | CP |
| 48 | 31 | male | Chronic pancreatitis | CP |
| 49 | 78 | female | Chronic pancreatitis | CP |
| 50 | 59 | female | Chronic pancreatitis | CP |
| 51 | 77 | male | Chronic pancreatitis | CP |
| 52 | 39 | male | Chronic pancreatitis | CP |
| 53 | 41 | male | Chronic pancreatitis | CP |
| 54 | 18 | female | Chronic pancreatitis | CP |
