# Supplementary material for: ATF6 regulates the development of chronic pancreatitis by inducing p53-mediated apoptosis
Source: Cell Death Dis. 2019 Sep 10;10(9):662. doi: 10.1038/s41419-019-1919-0 (PMC6737032; doi:10.1038/s41419-019-1919-0)
Supplement: Supplementary file 4 — Table S2 [file 41419_2019_1919_MOESM4_ESM.docx]

**Table 1. Primer sequences used for quantitative RT-PCR assay.**

| Primer ID | Sequences (5’-3’) |
| --- | --- |
| IL-1 F | TCAGGCAGGCAGTATCACTC |
| IL-1 R | AGCTCATATGGGTCCGACAG |
| IL-6 F | CTGCAAGAGACTTCCATCCAG |
| IL-6 R | AGTGGTATAGACAGGTCTGTTGG |
| TNF-F | GTACCTTGTCTACTCCCAGGTTCTCT |
| TNF-R | GTGTGGGTGAGGAGCACGTA |
| ATF4 F | GAAAGTTTAATAAAAGTCG |
| ATF4 R | AGTAATGTAAGCAGCAGAG |
| ATF6 F | CGCCTTTTAGTCCGGTTCTT |
| ATF6 R | CCAGTTGGTAACAATGCCATGT |
| XBP1 F | GTAGCAGCGCAGACTGCTCG |
| XBP1 R | GAACCTCGTCAGGATCCAGCGTG |
| CHOP F | ACCTTCACTACTCTTGACCCTG |
| CHOP R | GATGTGCGTGTGACCTCTGT |
| p53 F | GGCAGACTTTTCGCCACAG |
| p53 R | CAGGCACAAACACGAACCTC |
| -Actin F | GGCTGTATTCCCCTCCATCG |
| -Actin R | CCAGTTGGTAACAATGCCATGT |
