# Supplementary material for: ATF6 regulates the development of chronic pancreatitis by inducing p53-mediated apoptosis
Source: Cell Death Dis. 2019 Sep 10;10(9):662. doi: 10.1038/s41419-019-1919-0 (PMC6737032; doi:10.1038/s41419-019-1919-0)
Supplement: Supplementary file 5 — Supplementary Methods [file 41419_2019_1919_MOESM5_ESM.docx]

**Supplementary Methods**

***Establishment of PRSS1 transgenic mice, ATF6 knock out mice and ATF6 adenovirus***

Human trypsinogen PRSS1 (GenBank Accession Number: NM_002769.4) was specifically overexpressed in mice pancreatic acinar cells as follows. The genomic PRSS1 DNA sequences were first subcloned into the Hsp68-LacZ vector by the bacterial artificial chromosome technique. F0 transgenic mouse embryos were generated by pronuclear injection of gel-extracted lacZ reporter fragments (Figure S1A) (Cyagen).

The ATF6 gene (NCBI Reference Sequence: NM_001081304; Ensembl: ENSMUSG00000026663) is located on mouse chromosome 1. 16 exons were identified, with the ATG start codon in exon 1 and the TAG stop codon in exon 16 (Transcript: ENSMUST00000027974). Exon 2 were selected as knockout region. Deletion of this region resulted in the loss of function of the mouse Atf6 gene. Cas9, gRNA and targeting vector were co-injected into fertilized eggs for knockout mouse production (Figure 7A) (Cyagen).

Mice descendants were raised in a pathogen-free barrier facility, and those matched by sex and age were used for subsequent treatment and analysis. Single transgenic littermates undergoing the same treatments were applied as the controls. All mouse colonies were maintained in a specific pathogen-free barrier facility at the Nanfang Hospital laboratory animal center. PRSS1 genotyping was done by PCR using the following primers: PRSS1 forward: 5′-GAGCGGATTTGAACGTTGTG-3′; PRSS1 reverse: 5′-TACTTGAAGAGATTTGGCGG-3′; ATF6 forward: 5′-CTTGGCAGCACCTTGACCTTC-3′; ATF6 reverse: 5′-TGGCTCGGCAGGGAGAAATG-3′.

To investigate the effect of ATF6 on apoptosis and inflammation, primary acinar cells isolated from PRSS1 transgenic mice were infected with adenovirus vectors harboring full-length ATF6 for ATF6 overexpression, and a shATF6 fragment (generated siRNA: AACTCAGCACGTTCCTGAG) or scramble shRNA (scramble siRNA: CCTAAGGTTAAGTCGCCCTCG) negative control for ATF6 inhibition (Figure S1C). All adenovirus vectors were purchased from VectorBuilder Inc. (Guangzhou, China).

***CP induction and treatment***

To establish the CP animal model, male transgenic mice were treated with 15 μg/mL caerulein solution dissolved in phosphate-buffered saline by intraperitoneal injection, 50 μg/kg each hour for 8 h. Subsequent replicates of caerulein injection were shown in Figure S1B. Wide-type mice undergoing the same caerulein treatment were used as the control group. The development of CP symptoms was evaluated 12 h after caerulein injection. The ATF6 knockout PRSS1 transgenic mice were injected with adenovirus three days prior to caerulein injection. Virus suspension with a titer of 1×10^12^ was injected into the pancreatic tissue at a dose of 10-15 μl/mouse (mass 18-23 g). Five points of pancreatic tissue was selected for adenovirus injection each time. After the virus injection, caerulein injection was then performed subsequently. Transgenic mice were intraperitoneally injected with 2.2 mg/kg pifithrin-α (PFT-α) each day before the caerulein injection to inhibit p53 expression, followed by repeated PFT-α injections as shown in Figure S1B. Daily intragastrical injections of 0.5 g/kg tauroursodeoxycholic acid (TUDCA) was administered to transgenic mice after the first caerulein treatment to suppress the ER stress response. The transgenic mice were separately sacrificed 1, 2, and 4 weeks after the first caerulein injection.

For cell transfection with adenovirus, 1×10^12^ infectious units of adenovirus were added to primary acinar cells, which were co-cultured in 6-well plates one day prior virus infection. E eighteen hours after infection, cell culture medium with virus was replaced by fresh DMEM containing 10% fetal bovine serum (Invitrogen, Carlsbad, CA, USA).

***IHC and immunofluorescence assays***

Pancreatic tissues collected from patients, volunteers and mice were first sliced into 5-μm tissue sections, fixed at 4°C with 4% neutral phosphate-buffered formalin and embedded in paraffin. Hematoxylin and eosin (H&E) staining and IHC analysis of tissue slides were performed as previously described^34^. Briefly, a double-blind evaluation of tissue slides was carried out by experienced pathologists working in the Department of Pathology of Southern Medical University. The IHC assay based on the horseradish peroxidase system was performed using specific antibodies targeting ATF6 (Novus Biologicals; diluted 1:500), C/EBP-homologous protein (CHOP) (Abcam; diluted 1:200), XBP-1 (Abcam; diluted 1:500), collagen I (Thermo Fisher; diluted 1:500), a-SMA (Abcam; diluted 1:500), and p53 (Proteintech; diluted 1:100). IHC results were evaluated by scoring based on ratios of positively stained cells from at least three biological replicates. For simultaneous detection of p53 and a-SMA expression by IF, tissue slides were incubated with fluorescein isothiocyanate (FITC) or phycoerythrin (PE)-conjugated secondary antibodies (Sangon, Shanghai) after primary antibody incubation, and finally analyzed by fluorescent microscopy.

***Masson’s trichrome staining***

The collagen fiber content in pancreatic tissues was determined using the Masson's Trichrome Stain Kit (G1340; Solarbio Science & Technology, Beijing, China) following the manufacturer’s instructions. Briefly, tissue slides were first stained with Weigert's iron hematoxylin solution (Solarbio Science & Technology) for 5 min, incubated with a differentiation acidic ethanol solution for 10 sec and stained with Biebrich scarlet-acid fuchsin solution for 5 min. Subsequently, tissue slides were treated with a phosphomolybdic acid solution for 2 min, and the collagen components in the pancreatic tissues were stained with aniline blue for 1–2 min, followed by rinsing with acetic acid, dehydration with ethanol, clearing in xylene, and embedding with paraffin.

***Quantitative RT-PCR***

Quantitative RT-PCR was performed in this study to detect gene mRNA levels in mouse serum or pancreatic tissues. Briefly, total RNA samples from pancreatic tissues or serum were extracted using the TRIzol reagent (Thermo Fisher Scientific) following the manufacturer’s instructions. Approximately 3.0 μg total RNA samples were used for the synthesis of cDNA using the High Capacity cDNA Reverse Transcription Kit (Thermo Fisher Scientific) as instructed by the manufacturer. The relative gene mRNA levels were evaluated by quantitative RT-PCR using the QuantiFast SYBR Green PCR Kit (Qiagen) following the manufacturer’s instructions. Beta-actin was used as the internal standard for normalization. At least three biological replicates were examined. The sequences of primers used are listed in Table S2.

***Western blotting***

Total protein was extracted from the mouse pancreatic tissues using the Tissue or Cell Total Protein Extraction Kit (C510003; Sangon, Shanghai, China) following the manufacturer’s instructions. After protein concentration determination, approximately 25 μg protein from each sample was denatured at 100°C for 5 min, separated by 12% SDS-PAGE, and transferred onto a PVDF membrane (Millipore). After blocking with 5% lipid-free milk solution, the membranes were incubated with primary antibodies at room temperature for 1–2 h or 4°C overnight, washed three times with PBS solution for 5 min, and incubated with horseradish peroxidase-conjugated secondary antibodies for 2 h. The immunocomplexes were finally developed with enhanced chemiluminescence solution (Thermo Fisher Scientific). GAPDH was used as the internal standard. Primary antibodies used in this study include anti-ATF6 (Novus Biologicals), anti-CHOP (Abcam), anti-XBP-1 (Abcam), anti-p53 (Proteintech), and anti-GAPDH (Abcam). The protein abundance was evaluated by immunoblotting with at least three biological replicates.

***TUNEL assay***

Tissue slides embedded in paraffin were de-waxed with dimethylbenzene and hydrated with a routine ethanol gradient. Cell apoptosis in mouse pancreatic tissues was analyzed using the TUNEL method with a DNA Fragmentation Imaging Kit (QIA39; Sigma Aldrich) as instructed by the manufacturer. Green staining in the cell nucleus indicated apoptotic cells with DNA cleavage and fragmentation. DAPI was used to stain the nucleus for the localization of apoptotic cells. The TUNEL assay was performed with at least three biological replicates.
