# Supplementary material for: ATF6 regulates the development of chronic pancreatitis by inducing p53-mediated apoptosis
Source: Cell Death Dis. 2019 Sep 10;10(9):662. doi: 10.1038/s41419-019-1919-0 (PMC6737032; doi:10.1038/s41419-019-1919-0)
Supplement: Supplementary file 6 — Supplementary figure legends [file 41419_2019_1919_MOESM6_ESM.docx]

**Supplementary Figure Legends**

**Figure S1.** (A) Schematic diagram of ATF6 overexpression in PRSS1 transgenic mice. (B) Schematic presentation of chronic pancreatitis (CP) induction and treatment in PRSS1 transgenic mice overexpressing the human PRSS1 gene. The CP model mice were sacrificed at 1, 2, or 4 weeks for analysis.

**Figure S2.** Pancreas sizes in wild-type (WT) and PRSS1 transgenic mice treated with caerulein at 1, 2, and 4 weeks.
